# Supplementary material for: Mechanisms governing the pioneering and redistribution capabilities of the non-classical pioneer PU.1
Source: Nat Commun. 2020 Jan 21;11:402. doi: 10.1038/s41467-019-13960-2 (PMC6972792; doi:10.1038/s41467-019-13960-2)
Supplement: Supplementary file 7 — Source data [file 41467_2019_13960_MOESM7_ESM.zip › Source_Data/FigureS2/FigureS2B_MotifScanOutput/KmeansCluster4/homerResults/motif3.similar.html]

motif3

## Information for motif3

A
G
T
C
A
G
C
T
A
G
C
T
A
G
T
C
G
A
T
C
G
A
C
T
A
T
G
C
G
A
C
T
A
C
G
T
G
A
T
C
G
A
T
C
G
A
C
T
A
G
T
C
  
Reverse Opposite:  

T
C
A
G
C
T
G
A
C
T
A
G
C
T
A
G
T
G
C
A
C
T
G
A
T
A
C
G
C
T
G
A
C
T
A
G
T
C
A
G
T
C
G
A
T
C
G
A
T
C
A
G
  

|  |  |
| --- | --- |
| p-value: | 1e-358 |
| log p-value: | -8.257e+02 |
| Information Content per bp: | 1.631 |
| Number of Target Sequences with motif | 1753.0 |
| Percentage of Target Sequences with motif | 38.83% |
| Number of Background Sequences with motif | 5435.5 |
| Percentage of Background Sequences with motif | 13.00% |
| Average Position of motif in Targets | 100.4 +/- 50.4bp |
| Average Position of motif in Background | 97.4 +/- 88.5bp |
| Strand Bias (log2 ratio + to - strand density) | -0.1 |
| Multiplicity (# of sites on avg that occur together) | 1.40 |
| Motif File: | file (matrix) reverse opposite |

### Similar de novo motifs found

|  |  |  |  |  |  |  |  |
| --- | --- | --- | --- | --- | --- | --- | --- |
| Rank | Match Score | Redundant Motif | P-value | log P-value | % of Targets | % of Background | Motif file |
| 1 | 0.806 | G A C T A G T C G A T C G C A T A G T C G A C T G A C T A G T C G A T C G A C T A G T C G A T C G A C T A G T C | 1e-351 | -809.703617 | 62.39% | 31.60% | motif file (matrix) |
| 2 | 0.846 | T C G A A T C G C T G A T A C G C T A G G T C A C T G A T A C G C T G A C T A G C T A G G T C A | 1e-171 | -394.470134 | 11.47% | 2.12% | motif file (matrix) |
| 3 | 0.830 | A C T G C G T A C T A G C A T G G T C A G C T A A T C G G T A C G C T A C A T G T G C A C T G A T C A G T C G A | 1e-67 | -155.799130 | 8.44% | 2.76% | motif file (matrix) |
| 4 | 0.821 | C G T A A C T G C T A G C G T A C G T A A C T G C T G A C G T A A C T G C G T A C G T A A C T G | 1e-65 | -150.761245 | 3.26% | 0.38% | motif file (matrix) |
| 5 | 0.773 | C G T A A T C G C T G A A C T G C T A G C G T A T G C A A T C G C T G A C G T A T C A G C G T A C G T A | 1e-46 | -107.797547 | 3.52% | 0.73% | motif file (matrix) |
| 6 | 0.756 | C T G A A C T G C T A G C T G A A C T G C T A G C G T A A C T G C T A G C G T A | 1e-34 | -80.464455 | 3.26% | 0.84% | motif file (matrix) |
